# Supplementary figures and images for: Feeding preferences and the effect of temperature on feeding rates of the graceful kelp crab, Pugettia gracilis
Source: PeerJ. 2023 Apr 21;11:e15223. doi: 10.7717/peerj.15223 (PMC10124544; doi:10.7717/peerj.15223)

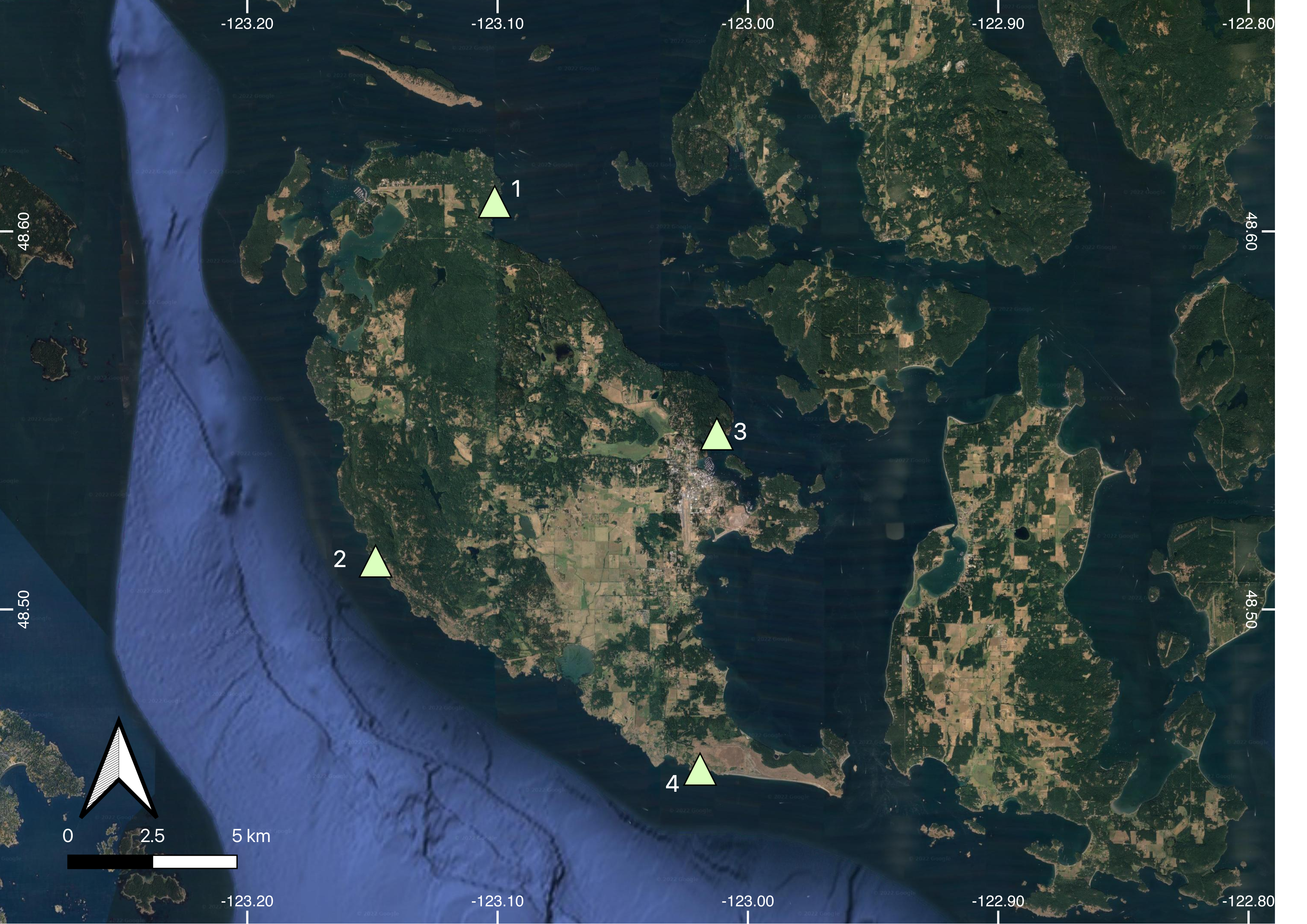

Supplement: Supplemental Information 1 — Location of four P. gracilis collection sites on San Juan Island, Washington. 1: Rueben Tart County Park (48°36′45″N, 123°05′53″W), 2: Deadman’s Bay (48°30′46″N, 123°08′47″W), 3: Friday Harbor Labs (48°32′43″N, 123°00′44″W), 4: Eagle Cove (48°27′39″N, 123°01′57″W). [file peerj-11-15223-s001.png]

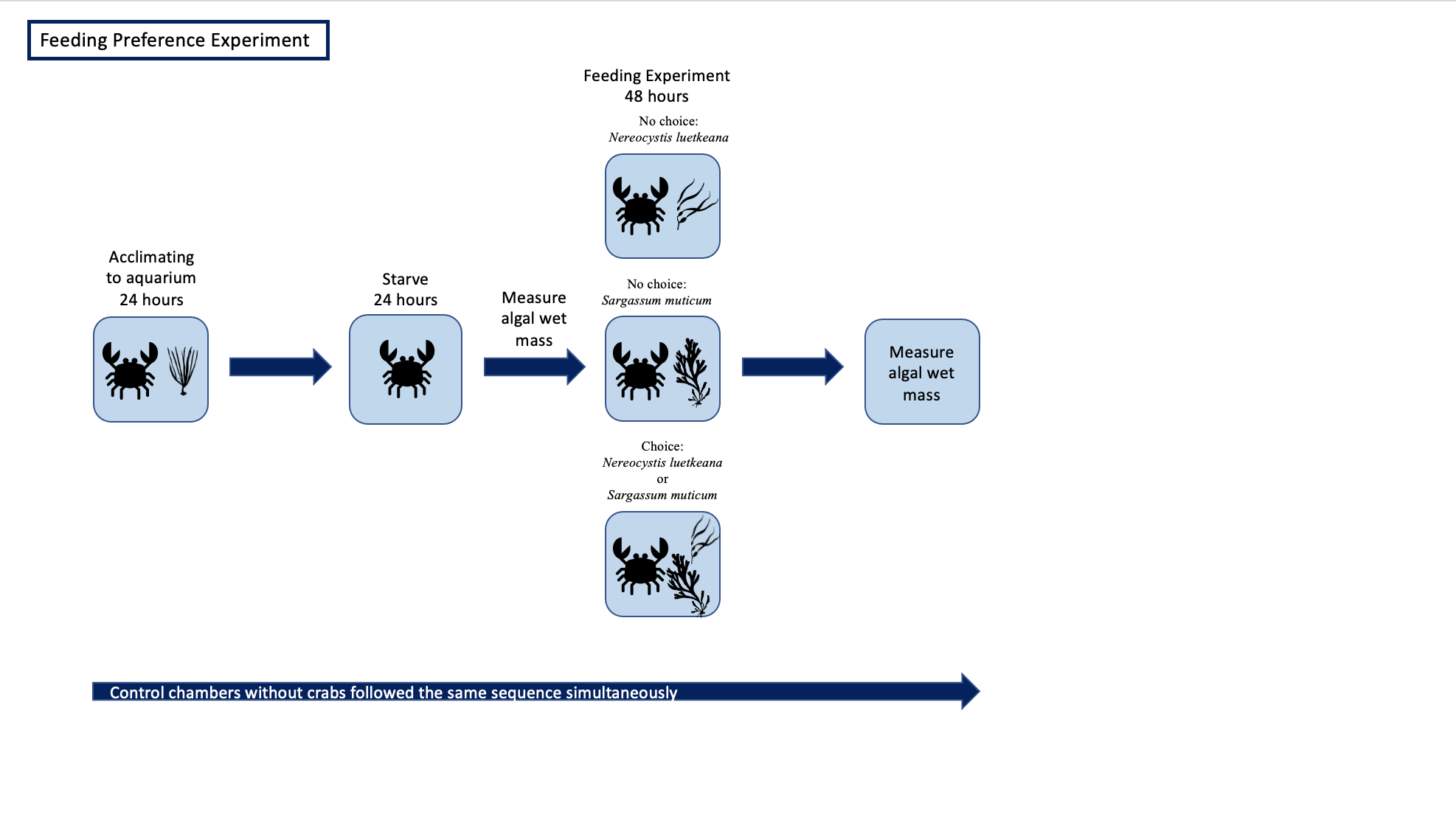

Supplement: Supplemental Information 2 [file peerj-11-15223-s002.png]

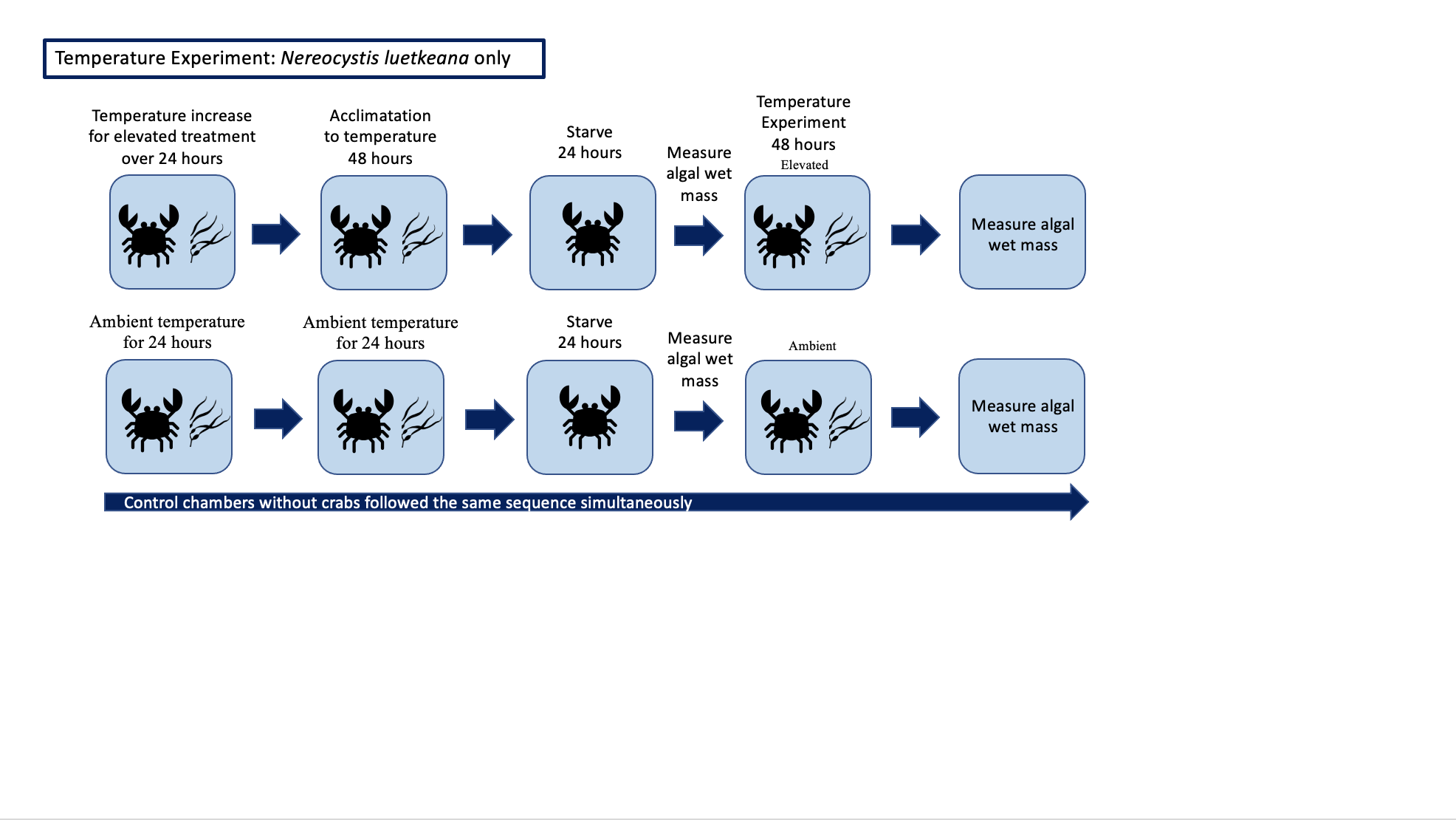

Supplement: Supplemental Information 3 [file peerj-11-15223-s003.png]
