# Supplementary material for: Feeding preferences and the effect of temperature on feeding rates of the graceful kelp crab, Pugettia gracilis
Source: PeerJ. 2023 Apr 21;11:e15223. doi: 10.7717/peerj.15223 (PMC10124544; doi:10.7717/peerj.15223)
Supplement: Supplemental Information 4 — Descriptive information regarding the crabs studied during feeding preference experiments; collection site refers to SI. [file peerj-11-15223-s004.docx]

| Crab | Sex | Crab Mass (g) | Temperature Treatment | Collection Site (Sup. Fig. I) |
| --- | --- | --- | --- | --- |
| 1B | Male | 1.8 | Ambient | 3 |
| 2B | Male | 3 | Ambient | 3 |
| 3B | Male | 2.3 | Elevated | 3 |
| 4B | Male | 2.2 | Ambient | 3 |
| 5B | Male | 2.7 | Elevated | 3 |
| 6B | Female | 2.8 | Ambient | 3 |
| 7B | Male | 3.4 | Ambient | 3 |
| 8B | Male | 3.7 | Ambient | 3 |
| 9B | Male | 4.8 | Elevated | 3 |
| 10B | Male | 4.6 | Ambient | 3 |
| 11B | Male | 3.1 | Elevated | 3 |
| 12B | Male | 3.6 | Ambient | 3 |
| 13B | Male | 3 | Ambient | 3 |
| 14B | Female | 3.2 | Elevated | 3 |
| 15B | Male | 2.3 | Ambient | 3 |
| 16B | Male | 2.2 | Ambient | 3 |
| 17B | Female | 5.8 | Ambient | 3 |
| 18B | Male | 5.6 | Elevated | 3 |
