# Supplementary material for: Feeding preferences and the effect of temperature on feeding rates of the graceful kelp crab, Pugettia gracilis
Source: PeerJ. 2023 Apr 21;11:e15223. doi: 10.7717/peerj.15223 (PMC10124544; doi:10.7717/peerj.15223)
Supplement: Supplemental Information 5 — Descriptive information regarding the crabs studied in the temperature dependent feeding trials; collection site refers to SI. [file peerj-11-15223-s005.docx]

| Crab | Sex | Crab Mass (g) | Collection Site (Sup. Fig. I) |
| --- | --- | --- | --- |
| 1A | Female | 1.3 | 2 |
| 2A | Male | 4.4 | 3 |
| 3A | Male | 5.8 | 3 |
| 4A | Male | 7.3 | 2 |
| 5A | Female | 3.4 | 1 |
| 6A | Male | 1.9 | 3 |
| 7A | Female | 2.7 | 2 |
| 8A | Male | 3.8 | 4 |
| 9A | Male | 3.4 | 2 |
| 10A | Female | 3.9 | 2 |
| 11A | Female | 1.2 | 2 |
| 12A | Female | 2.5 | 2 |
